# The Comparative Reliability and Feasibility of the Past-Year Canadian Diet History Questionnaire II: Comparison of the Paper and Web Versions

**DOI:** 10.3390/nu9020133

**Published:** 2017-02-13

**Authors:** Geraldine Lo Siou, Ilona Csizmadi, Beatrice A. Boucher, Alianu K. Akawung, Heather K. Whelan, Michelle Sharma, Ala Al Rajabi, Jennifer E. Vena, Sharon I. Kirkpatrick, Anita Koushik, Isabelle Massarelli, Isabelle Rondeau, Paula J. Robson

**Affiliations:** 1 Cancer Measurement, Outcomes, Research and Evaluation, CancerControl Alberta, Alberta Health Services, 1820 Richmond Rd SW, Calgary, AB T2T 5C7, Canada; Alianu.Akawung@ahs.ca (A.K.A.); Michelle.Sharma@ahs.ca (M.S.); Ala.Rajabi@ahs.ca (A.A.); Jennifer.Vena@ahs.ca (J.E.V.); 2 Departments of Oncology and Department of Community Health Sciences, Cumming School of Medicine, University of Calgary, Health Sciences Centre, Foothills Campus, 3330 Hospital Dr NW, Calgary, AB T2N 4N1, Canada; icsizmad@ucalgary.ca; 3 Prevention and Cancer Control, Cancer Care Ontario, 620 University Avenue, Toronto, ON M5G 2L7, Canada; Beatrice.boucher@cancercare.on.ca; 4 Department of Nutritional Sciences, Faculty of Medicine, University of Toronto, 150 College Street, Toronto, ON M5S 3E2, Canada; 5 Department of Health and Physical Education, Mount Royal University, 4825 Mount Royal Gate SW, Calgary, AB T3E 6K6, Canada; hwhelan@mtroyal.ca; 6 School of Public Health and Health Systems, University of Waterloo, 200 University Avenue West, LHN 1713, Waterloo, ON N2L 3G1, Canada; Sharon.Kirkpatrick@uwaterloo.ca; 7 Department of Social and Preventive Medicine, University of Montreal, and Centre de Recherche du CHUM (CRCHUM), 850 Saint-Denis Street, 2nd Floor, Montreal, QC H2X 0A9, Canada; Anita.Koushik@umontreal.ca; 8 Bureau of Food Surveillance and Science Integration, Food Directorate, Health Canada, Ottawa, ON K1A 0K9, Canada; Isabelle.Massarelli@hc-sc.gc.ca (I.M.); Isabelle.Rondeau@hc-sc.gc.ca (I.R.); 9 Alberta’s Tomorrow Project, CancerControl Alberta, Alberta Health Services, Sun Life Place, 15th floor, 10123 99 Ave, Edmonton, AB T5J 3H1, Canada; Paula.Robson@ahs.ca

**Keywords:** dietary assessment, feasibility, reliability, food frequency questionnaire

## Abstract

Advances in technology-enabled dietary assessment include the advent of web-based food frequency questionnaires, which may reduce costs and researcher burden but may introduce new challenges related to internet connectivity and computer literacy. The purpose of this study was to evaluate the intra- and inter-version reliability, feasibility and acceptability of the paper and web Canadian Diet History Questionnaire II (CDHQ-II) in a sub-sample of 648 adults (aged 39–81 years) recruited from Alberta’s Tomorrow Project. Participants were randomly assigned to one of two groups: (1) paper, web, paper; or (2) web, paper, web over a six-week period. With few exceptions, no statistically significant differences in mean nutrient intake were found in the intra- and inter-version reliability analyses. The majority of participants indicated future willingness to complete the CDHQ-II online, and 59% indicated a preference for the web over the paper version. Findings indicate that, in this population of adults drawn from an existing cohort, the CDHQ-II may be administered in paper or web modalities (increasing flexibility for questionnaire delivery), and the nutrient estimates obtained with either version are comparable. We recommend that other studies explore the feasibility and reliability of different modes of administration of dietary assessment instruments prior to widespread implementation.

## 1. Introduction

Among the various dietary assessment methods available (e.g., dietary records, 24-h dietary recalls, diet histories), food frequency questionnaires (FFQs) remain one of the most commonly-used tools in epidemiologic studies [1,2,3]. Although the error in data captured using FFQs has been highlighted [4,5], they remain a valuable tool in the dietary assessment of habitual intakes as they can be administered in a relatively simple, cost-effective, and time-efficient manner in very large numbers of people [4]. In particular, FFQs are useful for capturing intake of dietary components that may be consumed episodically among many in the population of interest [6,7]. Recent evidence suggests that administering FFQs in combination with another tool, such as 24-h recalls, may be of value, particularly in epidemiological research [8].

FFQs assess typical diet using predefined questions on frequency of consumption of food items over a specific period of time, and often include portion size [9]. In North America, the most widely used FFQs are the Block [10], the Willett [11] and the National Cancer Institute’s (NCI) Diet History Questionnaire (DHQ) [2,12]. The original NCI DHQ was updated in 2010, based on 24-h recalls (24HRs) collected in the US National Health and Nutrition Examination Surveys (NHANES) between 2001 and 2006. The revised version, known as the DHQ-II, consists of 134 items and 8 dietary supplement questions [13]. While many aspects of the U.S. and Canadian diets and food supplies are similar, some differences exist related to portion sizes consumed, fortification, and availability of specific food items and brands [14,15].

In light of these differences, both the DHQ-II and the companion nutrient database were modified for use in Canada by basing the food list and nutrient values on the Canadian Community Health Survey (CCHS)-Nutrition Cycle 2.2, 24-h dietary recall data [14]. New questions were added and existing questions modified to include items that contributed substantially to the intake of 17 nutrients. In total, 153 items were included in the CDHQ-II; of 143 items with portion sizes, 53 required portion size modification from the US DHQ-II to reflect Canadian consumption patterns. In addition, modifications were made to reflect the Canadian food supply, such as the inclusion of ethnic foods and expansion of the number of questions on dietary supplements.

Until recently, self-administered FFQs have been primarily paper-based and manually entered. Thus, errors due to skipped questions, complex skip patterns, missed responses and/or pages, multiple responses, and data entry are common [16,17]. In addition, paper-based FFQs may be relatively costly if administered in large studies, particularly when considering costs associated with mailing to and from participants, issuing reminders by mail or by phone, performing data entry and implementing extensive quality control procedures. In an attempt to reduce burden and costs, as well as errors, focus has shifted toward online technology [16,17], including the creation of a web version of the CDHQ-II suitable for administration in large-scale epidemiologic studies such as Alberta’s Tomorrow Project [18] and CARTaGENE [19], two large prospective cohort studies in Canada.

While administration and processing of the web CDHQ-II are likely to be less costly and burdensome than for the paper version, not all study participants may be willing or able to complete questionnaires online. For example, those living in rural areas or in places with poor internet coverage may find online questionnaire completion challenging [20]. Others may not have computer access or may prefer to complete paper questionnaires for reasons of privacy or convenience [20]. Differences in the structuring of the online and paper versions of the CDHQ-II raise questions about the relative ability to assess diet and the legitimacy of pooling data from questionnaires administered online and in paper versions within a single study [20]. Despite the assumed benefits of online FFQs, few studies have assessed the comparative reliability and/or validity of paper and web versions of FFQs [16,21,22,23] and no study has explored the reliability of different modes of administration of the CDHQ-II. Here, we evaluate the intra- and inter-version reliability, as well as feasibility and acceptability of repeated completions of both paper and web versions of the self-administered past-year CDHQ-II, in a sample of adults enrolled in Alberta’s Tomorrow Project.

## 2. Materials and Methods

### 2.1. Study Participants

Full details describing participant recruitment and enrollment in Alberta’s Tomorrow Project (ATP) are described elsewhere [18,24]. In brief, ATP is a longitudinal cohort of adults in Alberta that was launched in 2000 to provide a research platform enabling the study of the etiology of cancer and chronic disease. From 2000 to 2015, ~55,000 Albertans aged 35 to 69 years, able to complete written questionnaires in English, and with no personal history of cancer other than non-melanoma skin cancer at study initiation, were enrolled in ATP. This study was approved by the former Alberta Cancer Board’s Research Ethics Committee and the University of Calgary Conjoint Health Research Ethics Board (baseline data collection) and the Health Research Ethics Board of Alberta (Cancer Committee) (current analysis).

Sample sizes for the current study were estimated using methods described by Bonett et al. [25] for two-way mixed effects Analysis of Variance (ANOVA) models and R software, version 2.15.2 (R core team, Vienna, Austria). We estimated a sample size requirement of 273 to provide sufficient power to detect a minimum intra-class correlation coefficient (ICC) of 0.40 with a 95% confidence interval (CI) minimum total width of 0.20. Therefore, to evaluate the intra- and inter-version reliability of the paper and web versions of the CDHQ-II, we aimed to recruit 314 participants (273 plus 15% to account for possible attrition and data loss). Based on an estimated 50% response rate, 648 existing ATP participants who had provided email addresses (necessary to access the online CDHQ-II platform) were randomly selected, with balanced numbers of men and women, those aged <55 years and ≥55 years in order to include middle-aged and older participants, and those living in urban and rural areas. Selected participants were mailed invitation packages describing the feasibility study.

### 2.2. Study Design and Procedures

Recruitment and data collection took place between August 2014 and August 2015. Participants received an invitation package containing an invitation letter, consent form, and a short background questionnaire on smoking status, socio-demographic and anthropometric characteristics. Participants were considered enrolled if they returned the completed background questionnaire and signed consent form. Eligible participants who had not responded to the invitation package received a reminder by email at three weeks, a reminder postcard at six weeks, and one phone call at nine weeks to encourage study participation.

**Figure 1:**
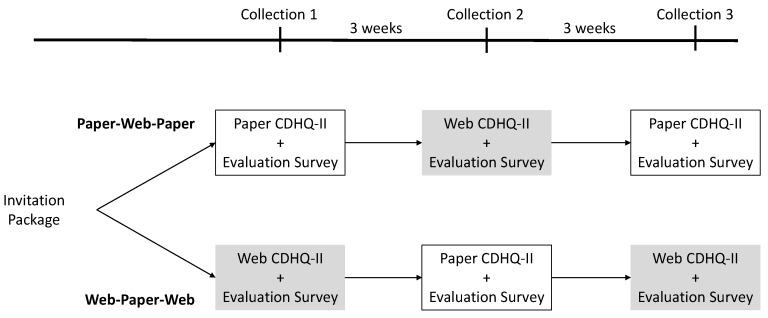
Study administration timeline and procedures for the Canadian Diet History Questionnaire II (CDHQ-II) paper and web feasibility study. CDHQ-II: Canadian Diet History Questionnaire II.

A simple randomization procedure was used to assign enrolled participants to one of two groups: (1) complete the CDHQ-II paper version first, followed by the web version, then paper again (Paper-Web-Paper); or (2) complete the web version first, followed by the paper, then web again (Web-Paper-Web). The randomization procedure involved generating a random number between 0 and 1 for each participant, with numbers less than 0.5 corresponding to the first group (Paper-Web-Paper) and numbers equal or greater than 0.5 corresponding to the second group (Web-Paper-Web). To reduce potential carry-over effects between completions of the two questionnaire versions but also avoid differences due to seasonal food availability and other sources of dietary change, a three-week washout period between the return of a completed questionnaire and the mailing of an invitation to complete the subsequent questionnaire was planned (Figure 1).

At Collections 1 and 3, a paper CDHQ-II was mailed to participants in the Paper-Web-Paper group, while a letter with instructions on how to access the web version as well as an email invitation with the URL for the web CDHQ-II were sent to participants in the Web-Paper-Web group. At Collection 2 (three weeks after receiving the completed first questionnaire), participants in the Paper-Web-Paper group were sent a letter and an email to access the web CDHQ-II, while participants in the Web-Paper-Web group were mailed the paper version. After mailing a paper CDHQ-II or letter with instructions on how to access the web version, participants who had not returned a questionnaire received a reminder by email at three weeks, and a phone call at six weeks.

At each collection, participants were mailed a short evaluation questionnaire that included questions pertaining to the feasibility and acceptability of the paper and web versions (Figure 1). The questionnaire asked about the length of time required to complete the CDHQ-II, willingness to complete the CDHQ-II online in the future (very willing, willing, unwilling, very unwilling), preference for the paper or web version, and also gave participants an opportunity to provide suggestions or additional comments about the CDHQ-II. The evaluation survey for the web version contained an additional 6 questions about internet connection type (dial-up, digital subscriber line (DSL), mobile 3G, public Wi-Fi (e.g., in a local coffee shop etc.), integrated services digital network (ISDN), cable modem, mobile 4G Long-Term Evolution (LTE), satellite, other), browser type and version (Internet Explorer, Safari, Google Chrome, Mozilla Firefox, Opera, other), if a public or private computer was used, as well as the computer operating system (MAC OS, Windows) and version. No specific questions about the content of the CDHQ-II were included in the evaluation questionnaire.

### 2.3. Canadian Diet History Questionnaire II (CDHQ-II)

English editions of the past-year CDHQ-II paper and web versions were used in the present study; full details describing this questionnaire are provided elsewhere [14]. Participants completing the web version followed automated skip patterns and had to complete all questions before answering the next question. Otherwise, contents of the CDHQ-II paper and web versions were identical with respect to questions. The paper version was scanned using Teleform software (Autonomy Company; Vista, CA, USA: Version 10.2) for automated optical scanning and data capture. The web version was created by the NCI (Bethesda, MD, USA) and Information Management Services Inc. (IMS) (Calverton, MD, USA). The IMS production computer centre resources are housed and operated within the Qwest CyberCenter in Sterling, VA, USA. Data from both the paper and web versions were analysed using Diet*Calc software, version 1.4.3 for Windows, (Canadian version, IMS, Calverton, MD, USA), and the CDHQ-II nutrient database was used to estimate mean daily intakes of energy and nutrients (food and beverage sources only, excluding supplement sources). The CDHQ-II paper and web versions are available from the NCI website [13,26].

### 2.4. Statistical Analysis

Descriptive statistics are presented as means (M), standard deviations (SD), medians and interquartile ranges (IQR) for continuous variables, and frequencies and percentages for categorical variables.

**Figure 2:**
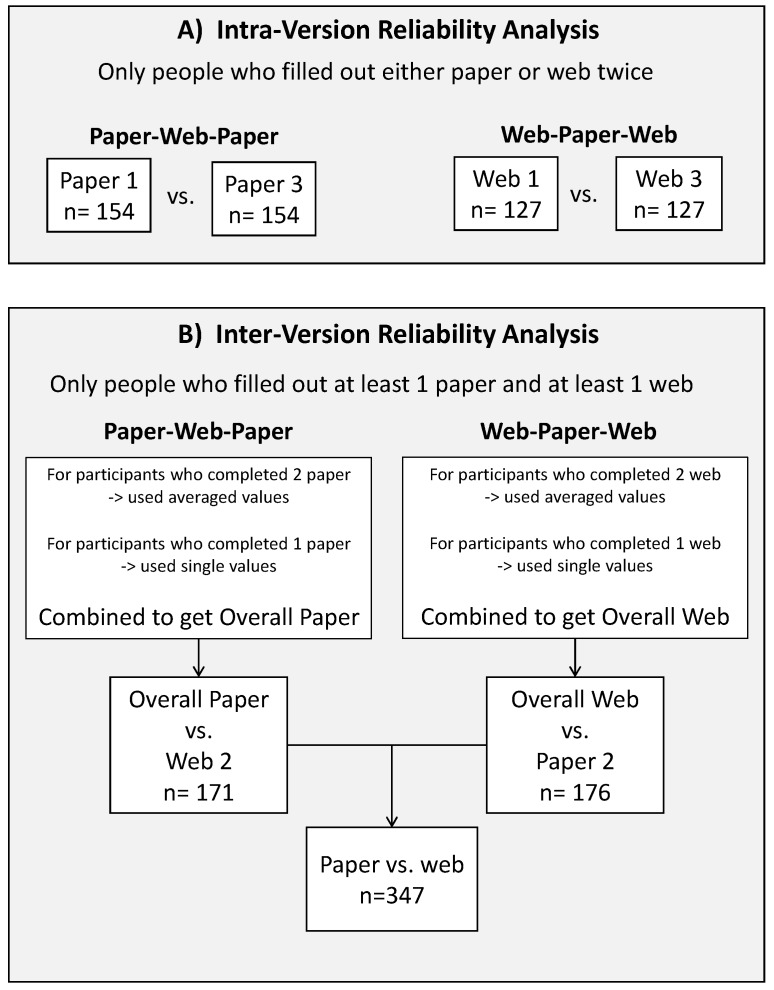
Schematic of the approach for the intra- and inter-version reliability analyses in the CDHQ-II feasibility study. CDHQ-II: Canadian Diet History Questionnaire II.

To assess the intra-version reliability of reporting for each CDHQ-II version, energy and 21 selected nutrient estimates were compared between the first and second completion in individuals who completed paper or web questionnaires twice (Collections 1 and 3; Figure 2); participants who completed a paper or web version at Collection 1, but did not complete a questionnaire at Collection 3 were excluded from the intra-version reliability analysis. Paired *t*-tests were used for comparisons for dietary components that were normally distributed (i.e., energy and selected nutrients contributed by food and beverage sources only) while Wilcoxon signed-rank tests were used for comparisons of data not satisfying the normal distribution assumption. Nutrient estimates were adjusted for total energy intake using the residual approach [27]. ICC with 95% CI were calculated for unadjusted and adjusted nutrient estimates, to assess agreement between paired observations, with values between 0.60 and 0.74 considered good, and with values between 0.75 and 1.00 considered excellent [28,29].

As few statistical differences were found in the intra-version reliability analysis, energy and nutrient estimates were averaged across the two paper questionnaires or the two web questionnaires (Collections 1 and 3) to create overall paper- or web-based energy and nutrient estimates in these respective groups for use in the analyses of inter-version reliability between paper and web versions. Averaged nutrient estimates from Collections 1 and 3 were then compared with estimates from the other version of CDHQ-II completed by the same participants at Collection 2 (i.e., the two paper collections were combined and averaged for comparison to the single web collection in the Paper-Web-Paper group, and vice versa for the Web-Paper-Web group). For participants who completed only one paper or web version, the nutrient estimates from their single paper or web completion were used in the inter-version reliability analysis. The two groups (Paper-Web-Paper and Web-Paper-Web) were then combined for an overall inter-version reliability analysis between paper and web CDHQ-II. Similar to the intra-version reliability analysis, paired *t*-tests, Wilcoxon signed-rank tests, and intra-class correlation coefficients (ICCs) (for unadjusted and adjusted nutrient estimates) were used in the inter-version reliability analysis. In addition, Bland-Altman plots [30,31] were constructed for visual assessment of agreement between the paper and web versions of the CDHQ-II.

Intakes of dietary supplements were analysed as binary variables (yes/no) and the kappa statistic [32] was used to assess the level of agreement between the paper and web versions. Finally, a 2 × 3 mixed ANOVA was used to assess time and group effects. Analyses were conducted separately for men and women using SPSS version 19.0 (IBM Corporation, Armonk, NY, USA) for Windows, and the criterion for statistical significance was set as alpha ≤0.05 (two-tailed).

Feasibility and acceptability of the paper and web versions were analysed descriptively using responses obtained from the evaluation surveys. Responses to questions about future willingness to complete the CDHQ-II online were combined in two categories: (1) very willing and willing; and (2) unwilling and very unwilling. Responses to questions about type of internet connection were combined into three categories: low speed (dial-up, mobile 3G, ISDN); medium speed (public Wi-Fi, mobile 4G LTE, satellite) and high speed (DSL, cable modem). The paired *t*-test was used to assess differences in time spent completing the paper and web versions, while the chi-square test was used to assess differences in future willingness to complete the CDHQ-II online, type of internet browsers, internet connection method, computer operating system, paper and web preferences across sex, age, education, and geographic location.

## 3. Results

### 3.1. Study Participants

**Figure 3:**
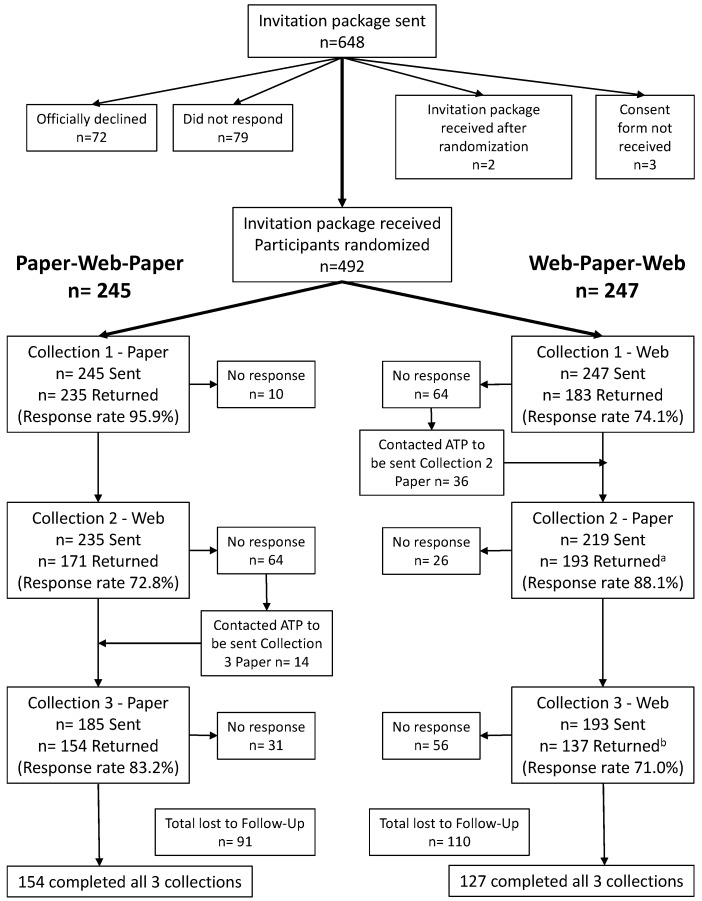
Participant flow in the CDHQ-II Feasibility study. ATP: Alberta’s Tomorrow Project; CDHQ-II: Canadian Diet History Questionnaire II. ^a^ 17 participants in the Web-Paper-Web group did not complete a web questionnaire at Collections 1 and 3 but returned a paper questionnaire at Collection 2; ^b^ 10 participants did not complete a web questionnaire at Collection 1 but returned a web questionnaire at Collection 3.

Of the 648 invited participants, 72 declined to participate in the study, 79 did not respond, three returned the background questionnaire but not the consent form, and two returned both the background questionnaire and consent form after randomization had been completed and thus were not enrolled. A final sample of 492 enrolled participants was randomized between the two groups. Unless otherwise specified, only participants who completed the CDHQ-II at the previous collection point were sent the invitation at Collections 2 and 3. More specifically, among the 64 participants of the Paper-Web-Paper group who did not complete the web CDHQ-II at Collection 2, 14 indicated they wanted to complete the paper at Collection 3. Similarly, among the 64 participants of the Web-Paper-Web group who did not complete the web CDHQ-II at Collection 1, 36 indicated they wanted to complete the paper at Collection 2 (Figure 3).

Response rates decreased from 96% to 83% in the Paper-Web-Paper group and 74% to 71% in the Web-Paper-Web group over the three collection time points. Overall, 57.1% of 492 participants completed all 3 collections.

A total of 582 of 649 paper CDHQ-II were returned, giving an overall paper response rate of 89.7% over all collection points and questionnaire groups; while a total of 491 of 675 web CDHQ-II were returned, giving an overall web response rate of 72.7% (*p* < 0.001). Although 3 weeks between completions was planned, the mean (±standard deviation (SD)) length of time between Collection 1 and 2 questionnaires, and Collection 2 and 3 questionnaires, was 7.0 (±3.5) and 7.0 (±4.3) weeks for the Paper-Web-Paper group, and 7.3 (±4.5) and 6.4 (±3.0) weeks for the Web-Paper-Web group. Finally, we examined results with the exclusion of participants with energy intakes assumed to be biologically implausible (<800 kcal/day or >4200 kcal/day for men (*n* = 13) and <600 kcal/day or 3500 kcal/day for women (*n* = 7)) [2], but as this exclusion had no significant impact on our results, all participants were retained for analyses.

### 3.2. Participant Characteristics at Enrollment

Participant characteristics of men and women at enrollment are presented in Table 1.

The mean (±SD) age of participants was 56.9 (±8.8) years and mean (±SD) body mass index (BMI) was 27.3 (±4.9) kg/m^2^. The majority reported living with a partner (88.0%) and were non-smokers (94.7%). While 79.2% of men were classified as having a BMI ≥ 25 kg/m^2^, only 54.6% of women were classified as overweight or obese. However, the majority of men reported waist circumference measurements in the low risk category (<102 cm), in contrast to a majority of women who reported measurements in the high risk category (>88 cm).

### 3.3. Intra-Version Reliability of Paper and Web CDHQ-II

In men, significant differences in mean intakes between Collections 1 and 3 were found for percent energy from carbohydrates, percent energy from total fat, carbohydrates, dietary fibre, caffeine, sodium, iron and total folate in the Paper-Web-Paper group, with most mean intakes significantly lower at the second time point (Table 2). In women, no significant differences in the mean intakes between Collections 1 and 3 were found except for total folate, which was lower at the second time point. With the exception of cholesterol, and vitamin B_12_ for men and polyunsaturated fat for women, ICCs for adjusted nutrients were ≥0.60 in both sexes, indicating good similarity between the two paper version completions in the Paper-Web-Paper group. Adjusting for energy intake reduced the ICCs when compared to the ICCs for unadjusted nutrient intakes (Supplementary Materials Table S1); however, a majority of nutrients had ICCs ≥ 0.60 for adjusted and unadjusted intakes.

In men, no significant differences in mean intakes of energy or nutrients were observed between Collections 1 and 3 for the two web version completions in the Web-Paper-Web group (Table 3). In women, significantly lower mean intakes were found at the second web completion for total energy, carbohydrates, dietary fibre, polyunsaturated fat, iron, calcium and total folate. With the exception of polyunsaturated fat, sodium, calcium and vitamin B_12_ in men and carbohydrates, total sugars, monounsaturated fat, and sodium in women, ICCs for adjusted nutrients were ≥0.60 in both sexes, indicating good similarity between the two web version completions in the Web-Paper-Web group. As observed with the paper version, adjusting for energy intake reduced the ICCs when compared to the ICCs for unadjusted nutrient intakes (Supplementary Materials Table S1); however, a majority of nutrients had ICCs ≥ 0.60 for adjusted and unadjusted intakes.

### 3.4. Inter-Version Reliability of Paper and Web CDHQ-II

Table 4 provides an overview of the inter-version reliability between the paper and web CDHQ-II, assessed by comparing energy and 21 selected nutrient estimates from the two versions completed by the same participants (Table 4).

In men, no significant differences were observed in mean intakes based on paper and web versions. In women, significant differences in the means between paper and web versions were found for total energy, carbohydrates, dietary fibre, monounsaturated fat and protein; all estimates were higher in the web version. With the exception of vitamin B_12_ in men and women, ICCs for adjusted nutrients were ≥0.60 in both sexes, indicating good similarity between the paper and web versions.

High agreement in reporting dietary supplement intake was found between paper and web versions (Supplementary Materials Table S2). In addition, there were no significant time and group effects for energy and 21 selected nutrients assessed in the mixed ANOVA (Supplementary Materials Table S3).

**Figure 4:**
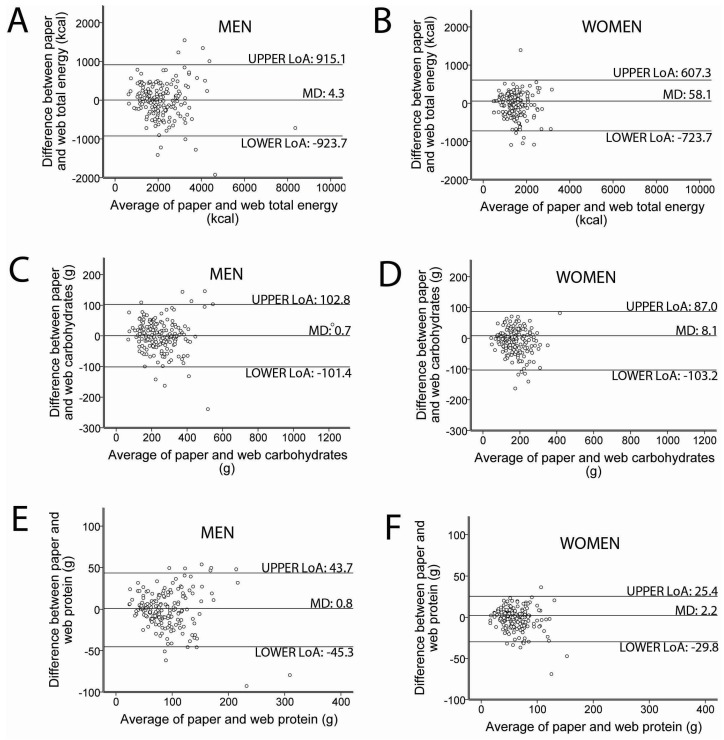
Bland-Altman plots depicting total energy, carbohydrates and protein intake between paper and web CDHQ-II. (**A**) Total energy intake for men; (**B**) Total energy intake for women; (**C**) Carbohydrates for men; (**D**) Carbohydrates for women; (**E**) Protein for men; (**F**) Protein for women. CDHQ-II: Canadian Diet History Questionnaire II, LoA: Limits of Agreements, MD: Mean Difference. The plots show the 95% LoA between estimates of total energy, carbohydrates and protein intake from paper and web CDHQ-II in men (*n* = 176) and women (*n* = 171).

Representative Bland-Altman plots are shown for total energy intake, carbohydrates and protein, in men and women (Figure 4). Mean differences in the selected nutrients were close to zero. The widths between upper and lower limits of agreement were relatively wide for energy (1331 kcals for women, 1839 kcals for men) suggesting somewhat limited agreement between the paper and web CDHQ-II; however, the narrower ranges observed for other nutrients and in women compared to men suggested better agreement.

### 3.5. Feasibility and Acceptability

The majority of participants in both Paper-Web-Paper and Web-Paper-Web groups indicated future willingness to complete the CDHQ-II online at each collection (>89%, Supplementary Materials Table S4). In both groups, virtually all (>99%) of the participants with elementary school education reported future willingness to complete the CDHQ-II online at all collection points, except for Collection 1 in the Paper-Web-Paper group (75%). In addition, at least 84% of participants with higher educational attainment in both groups reported such willingness at all collection points. Furthermore, in both groups and at all collection points, a higher proportion of participants aged <55 years (>90% vs. >88%) reported future willingness to complete the CDHQ-II online, except for Collection 3 in the Web-Paper-Web group. Finally, for both groups and at all three collections, a higher proportion of participants living in urban areas reported future willingness to complete the CDHQ-II online, compared to those living in rural areas (>90% vs. >84%).

When participants were asked whether they preferred paper or web versions, 59% (52.1% in the Web-Paper-Web group and 65.6% in the Paper-Web-Paper group) indicated they preferred the web CDHQ-II at both Collections 2 and 3. A few (9.0%) participants in the Paper-Web-Paper group and 20.2% of participants in the Web-Paper-Web group changed their preference between Collections 2 and 3 to match the CDHQ-II version they had just completed. In contrast, only 2.3% of participants in Paper-Web-Paper group and 0.8% participants in Web-Paper-Web group changed their preference between Collections 2 and 3 to choose the CDHQ-II version that was not assigned to them at the corresponding collection point.

Analyses of the time required for CDHQ-II completion was restricted to participants who completed the evaluation survey at all collections. When combining responses from both groups (*n* = 206), mean (±SD) completion time for the paper version (83 ± 40 min) was higher than the web version (77 ± 32 min). Completing the same CDHQ-II version twice did not affect time required for completion, as the completion time at Collection 1 was not different from Collection 3 for either paper (86 ± 49 vs. 83 ± 42) or web versions (76 ± 31 vs. 74 ± 29). Completion time for the paper version was consistently higher than the web version regardless of sex, age categories, educational level, and geographic location.

The time spent completing and future willingness to complete the CDHQ-II online by type of browser, internet connection and operating system are summarized in Table 5. At any given collection point, most participants who completed the web CDHQ-II reported using high speed internet and a Windows operating system, while Internet Explorer was the most frequently used browser. Significant differences in time spent completing the web CDHQ-II by types of browser and internet connection were not observed. Similarly, significant differences in the proportion of participants willing to complete the CDHQ-II online in the future by types of browser, internet connection and operating system were not observed.

Finally, statistically significant differences in the proportion of participants in the Paper-Web-Paper group who dropped out of the study between Collections 2 and 3 were found across type of internet connection (low speed 5.3%, medium speed 26.5% and high speed 9.0%, *p* = 0.017). In the Web-Paper-Web group, statistically significant differences in the proportion of people who dropped out between Collections 1 and 2 (*n* = 28) were found across browser type (Internet Explorer 14.9%, Safari 3.2%, Google Chrome 2.0% and Mozilla Firefox 20.0%, *p* = 0.029).

## 4. Discussion

Findings from this study support intra- and inter-version reliability at the group level of the paper and web versions of the past-year CDHQ-II, in this sub-group of participants enrolled in an existing cohort study. With few exceptions, the findings suggest that the paper and web versions produce comparable estimates of energy and nutrient intakes. In the present study, the majority of participants indicated future willingness to complete the CDHQ-II online. When asked about their preference between the paper and the web CDHQ-II, 59% of participants indicated a preference for the web version. Further, the web CDHQ-II was feasible and acceptable among this adult cohort, suggesting this modality may be useful for deployment in very large epidemiological studies in which it is necessary to limit cost and administrative burden.

Few studies have examined the reliability of dietary intake estimates from self-administered paper vs. web versions of other FFQs. Beasley et al. [22] reported variable reliability correlations for various nutrients, ranging from 0.60 to 0.81 (unadjusted for energy) and 0.28 to 0.73 when adjusted for energy, based on data from 210 adults who completed the paper DHQ-I and a web pictorial diet history questionnaire (Web-PDHQ) that was developed by modifying the DHQ-I to include portion size photographs. Using a cross-over design, González Carrascosa et al. [23] compared nutrient intakes from the past-year paper version of an FFQ to the online version in a sample of 39 university students, reporting correlation coefficients (unadjusted for energy) that ranged from 0.18 to 0.70 (median *r* = 0.47) and differed significantly between paper and online versions for all nutrients but fibre [23]. The authors concluded that these differences may be partly attributable to the absence of portion size photographs in the paper FFQ. Boeckner et al. [21] reported correlation coefficients (unadjusted for energy) in the range of 0.54 to 0.86 (median *r* = 0.80) for all nutrients examined when comparing the paper version of the 1998 Health Habits and History Questionnaire (HHHQ) to a web version of the same FFQ in a sample of 29 women, concluding adequate reliability of the two versions. Finally, although Kristal et al. [16] did not compare with a web FFQ, the authors reported correlations (adjusted for energy) ranging from 0.49 to 0.87 for nutrients examined when comparing two administrations of the Graphical Food Frequency System (GraFFS), a past three-month web-based FFQ, in a sample of 74 participants.

Our ability to draw comparisons with other reliability studies is somewhat limited, as correlation coefficients for adjusted nutrients have been reported in few studies, including those by Beasley et al. [22] and Kristal et al. [16]. For the inter-version reliability analysis in this study, ICCs for adjusted nutrients were somewhat higher than those reported by Beasley et al. [22]; whereas the ICCs for unadjusted nutrients are in line with Beasley et al. [22] and Boeckner et al. [21], but higher than those reported by González Carrascosa et al. [23]. The comparison of Web-PDHQ vs. paper DHQ-I in Beasley et al. [22] was conducted on 210 participants (mean ages 54.9 years), and since Beasley et al. [22] used the DHQ-I (the FFQ the CDHQ-II was adapted from), the higher ICCs observed in the present study may partly be attributed to using non-pictorial paper and web CDHQ-IIs. For the intra-version reliability analysis of this study, the ICCs for adjusted nutrients were consistent with those reported by Kristal et al. [16]; whereas the ICCs for unadjusted nutrients were in line with Beasley et al. [22], and this may partly be attributed to using the same questionnaire in the same modality and the long reference period for CDHQ-II (i.e., past year), DHQ-I (i.e., past year), and GraFFS (i.e., past three months).

In addition, there are challenges in comparing different studies with different sample sizes. The sample size in González Carrascosa et al. [23] was much lower than the present study (39 vs. 347), and participation was restricted to university students with a mean age of 24.8 years, representing a much younger population than the ATP cohort (mean age 56.9 years). Hence, the differences in the correlation coefficients may be partly attributed to differences in the reporting characteristics of the two populations in addition to differences in the tools (i.e., an FFQ with 153 items in the present study vs. a substantially shorter list of 84 items in the earlier study). Although the sample size in Boeckner et al. [21] was also much lower than the present study (29 vs. 347), the mean ages were very similar (58.2 years vs. 56.9 years) and the Boeckner FFQ listed almost as many food items (*n* = 121) [21] as our study, and included pictures of serving sizes in both the paper and web FFQ. The similarity in the correlation coefficients may be partly attributed to the fact that both studies involved participants in the same age group who might encounter similar issues completing the online version vs. the paper version, as well as the use of more comprehensive FFQs that are presumably better able to capture dietary intake.

The response rates overall and at each collection were higher for the paper CDHQ-II compared to the web version. This might be due to the assumed completion ease and portability of the paper FFQ, as well as undocumented technical issues encountered by some participants in completing the web version. It is also conceivable that ATP participants are more comfortable completing a paper questionnaire. Since its inception, ATP has captured information using paper questionnaires and has only recently started to transition to online data collection. Some participants identified a lack of high speed internet and eventually withdrew from the study, noting that many rural areas do not have accessible high speed internet, either due to high cost or lack of availability. Alberta is a large province with about 17% of people living in rural and remote locations [34]. As a result, this group of participants might have limitations in completing the web CDHQ-II. Several participants in the Web-Paper-Web group indicated they couldn’t complete the web CDHQ-II at Collection 1 due to technical issues, which may have been the case with other participants who did not respond but also did not formally withdraw from Collection 1 or the study overall. Almost all participants indicated they would be willing to complete the web CDHQ-II in the future, with a higher percentage among urban vs. rural participants, and 59% indicated they preferred the web CDHQ-II. A similar question on willingness was not asked for the paper version; hence, no comparison can be made. However, some participants said they would have to switch internet providers to complete online questionnaires in the future as their current internet service was too slow. Others reported they preferred the paper version because they felt their computer literacy was not sufficient for the web version. Participants who preferred the web version also mentioned environmental reasons.

Overall, the mean time spent completing the CDHQ-II using the web version was on average 6 min shorter than for the paper version. Time spent completing the CDHQ-II was lower for the web version than for the paper version across age categories, educational attainment and geographic location. Based on comments from the participants, the reduced time required to complete the web version may be attributed to ease of use (i.e., clicking boxes online compared to shading boxes on paper). In terms of technical requirements, overall the type of browser did not make a difference as long as a high-speed internet connection was available. On the other hand, despite some advantages of using web FFQs for dietary assessment, the web CDHQ-II has some limitations; as noted, certain populations, such as people who reside in remote areas, may not have access to high-speed internet and may not respond. We also noted an overall lower response rate for the online CDHQ-II than the paper version. This observation needs to be explored further in future studies, as it may necessitate a more ambitious reminder process than needed for paper questionnaires. Alternatively, it may be necessary to provide more resources to support participants in the transition to online data collection. The present study supports high intra- and inter-version reliability at the group level of the paper and web CDHQ-II, in this sub-group of ATP participants. Although previous studies have been conducted to assess the validity of the original NCI DHQ [2,12,35,36], no studies have been conducted specifically to assess the validity of the CDHQ-II. Validation of dietary assessment instruments is extremely complex and challenging, requiring evaluation against a marker of “truth”. Typically, this entails the use of recovery biomarkers, such as doubly labelled water (for energy expenditure) and 24 h urinary nitrogen (for protein), potassium, and sodium. Currently, there are no established biomarkers for food groups or nutrients other than energy, protein, potassium, and sodium, so it is not possible to use this approach to evaluate the CDHQ-II for the range of variables described in the present study. Nonetheless, the methods used to identify the food list, establish portion sizes and to generate the companion nutrient database for CDHQ-II were similar to those described for the original NCI DHQ by Subar et al. [37]. Therefore, previous findings of studies evaluating the original NCI DHQ are assumed to reflect the validity of the CDHQ-II.

Since data from self-reported dietary tools are subject to mis-reporting and other sources of measurement error, they have been recently criticized [38]; and Subar et al. recommended that self-reported energy intake should not be used as a measure of true energy intake [39]. Nonetheless, self-reported dietary intake data still contain valuable, rich, and critical information that can be useful to nutrition policy and to assess associations between diet and disease [39]. Statistical methods continue to be developed to correct for some of the systematic error associated with self-report. One promising approach suggests the use of FFQs in combination with short-term instruments, such as 24HRs, in an attempt to maximize strengths of both instruments and improve the risk estimates derived from studies of associations between diet and health.

The present study has other limitations that should be borne in mind. First, ATP participants represent existing members of an ongoing cohort; therefore, our participants may have been more motivated to complete the study requirements as opposed to a sample recruited from the general population. It must also be noted that we selected participants on the assumption that possession of an email address (74% of ATP participants) was indicative of having access to a computer connected to the internet, and that the quality of the connection would be adequate to support completion of a lengthy food frequency questionnaire. In practice, this may not have been a safe assumption. Indeed, our results suggest that logistics around the use of newer technologies should be checked rather than assumed in studies that intend to deploy new, electronic methods for dietary assessment.

In addition, although a three-week washout period between collections was planned, the average length of time between each collection was about 7 weeks in both Paper-Web-Paper and Web-Paper-Web groups. The washout period was aimed to be long enough to reduce potential carry-over effects between collections and short enough to avoid differences associated with seasonal food availability and other factors that might elicit dietary change. Given the study design, the time periods for each collection did not overlap; thus, any differences observed may be driven by fluctuations in diet rather than differences between the CDHQ-II versions. Nonetheless, since the CDHQ-II was designed to assess past year diet, differences due to seasonality and dietary change are unlikely to be substantial. Finally, using correlation coefficients to assess reliability of an FFQ, as was done in this study and previous studies [16,21,22,23], has limitations as both true intake and measurement error are correlated [40], potentially affecting the resulting estimates.

## 5. Conclusions

This study demonstrates that the paper and web versions of CDHQ-II are highly reliable at the group level in this sub-group of ATP participants, indicating that these two versions of CDHQ-II are likely to produce comparable results that may be combined. Although we observed some practical limitations and technical issues associated with deploying the web CDHQ-II in our study population, there remain numerous practical advantages, including remote administration, cost-efficiency, and reduced time to complete nutrient analyses [17,22], albeit with the understanding that electronic means of data capture may not be suitable for all participants. Thus, we recommend that studies aiming to combine dietary data obtained using the same instrument administered via different modalities should explore differences and similarities in data structure before embarking on widespread implementation across all participants.

## Figures and Tables

**Table 1:** Baseline characteristics of the study participants (*n* = 492).

Characteristics	Category	All Participants (*n* = 492) %	Men (*n* = 242) %	Women (*n* = 250) %
Age (Years)	<55	47.2	48.8	45.6
	≥55	52.9	51.2	54.4
Marital status	No partner ^a^	12.0	9.1	14.8
	With a partner ^b^	88.0	90.9	85.2
Employment status	Not employed ^c^	13.0	6.6	19.2
	Retired	22.6	18.6	26.4
	Employed part-time	9.4	3.7	14.8
	Employed full-time	55.1	71.1	39.6
Educational attainment	Elementary school	3.3	5.0	1.6
	High school completed	24.2	23.1	25.2
	Some post-secondary ^d^	46.3	45.5	47.2
	Post-secondary completed ^e^	26.2	26.5	26.0
Tobacco	Smoker ^f^	5.3	7.4	3.2
	Non-smoker ^g^	94.7	92.6	96.8
Body mass index (kg/m^2^) ^h^	<18.5	0.8	0.4	1.2
	18.5–24.9	32.5	20.3	44.2
	25.0–29.9	43.3	56.4	30.5
	≥30.0	23.5	22.8	24.1
Waist circumference ^i^	<102 cm (men); <88 cm (women)	47.5	64.1	31.3
	≥102 cm (men); ≥88 cm (women)	52.6	36.0	68.7
Geographic location ^j^	Rural	51.8	53.3	50.4
	Urban	48.2	46.7	49.6

^a^ No partner includes combined responses to: single, never married, divorced, separated, and widowed; ^b^ With a partner includes combined responses to: married and/or living with someone; ^c^ Not employed includes: looking after home and/or family, unable to work because of sickness or disability, unemployed, doing unpaid or voluntary work, and student; ^d^ Some post-secondary includes: Trade, technical or vocational school, apprenticeship training or technical collège d’enseignement général et professionnel (CEGEP), diploma from community college, pre-university CEGEP or non-university certificate, and university certificate below Bachelor’s level; ^e^ Post-secondary completed includes: Bachelor’s, and graduate degree (MSc, MBA, MD, PhD etc.); ^f^ Smoker includes: current daily (at least one cigarette every day for the past 30 days) and current occasional smoker (at least one cigarette in the past 30 days, but not every day); ^g^ Non-smoker includes: never and former smoker (did not smoke at all in the past 30 days); ^h^ Body mass index was categorized based on Health Canada’s classification scheme [33]; ^i^ Waist circumference was dichotomized using a cut-off of 102 cm for men and 88 cm for women [33]; ^j^ Geographic location was based on Canada postal codes where the second digit was 0 for rural regions.

**Table 2:** Summary of energy and nutrients ^a^ for the two paper CDHQ-II completions obtained at Collections 1 and 3 in the Paper-Web-Paper group, for men and women (*n* = 154).

Nutrient	Men (*n* = 72)				Women (*n* = 82)			
	Mean ± SD, Median (IQR)		Mean Difference ± SD	ICC ^b^ (95% CI)	Mean ± SD, Median (IQR)		Mean Difference ± SD	ICC ^b^ (95% CI)
	Paper Collection 1	Paper Collection 3			Paper Collection 1	Paper Collection 3		
Total energy (kcal)	2066 ± 848	1975 ± 757	91 ± 507	N/A	1550 ± 500	1522 ± 527	−28 ± 407	N/A
	1865 (919)	1857 (999)			1487 (614)	1425 (621)		
% energy carbohydrates	47.7 ± 7.9	46.3 ± 7.5	−1.4 ± 4.8 *	N/A	47.1 ± 6.7	46.5 ± 6.5	−0.7 ± 4.3	N/A
	48.5 (12.1)	47.4 (11.5)			46.2 (9.7)	46.3 (7.0)		
% energy total fat	34.4 ± 6.5	35.7 ± 6.4	1.3 ± 4.6 *	N/A	36.0 ± 5.7	36.4 ± 5.1	0.4 ± 3.7	N/A
	33.9 (6.6)	35.2 (6.7)			35.6 (6.7)	36.5 (5.9)		
% energy protein	16.1 ± 2.8	16.5 ± 3.0	0.4 ± 2.1	N/A	16.8 ± 3.1	17.1 ± 3.2	0.3 ± 2.2	N/A
	15.9 (3.2)	15.9 (3.0)			16.8 (3.5)	17.3 (3.4)		
Carbohydrates (g)	241 ± 89	225 ± 83	−15 ± 56 *	0.78 (0.67, 0.85)	182 ± 62	176 ± 61	−6 ± 47	0.72 (0.60, 0.81)
	227 (115)	215 (125)			182 (76)	170 (80)		
Total sugars (g)	104 ± 44	98 ± 38	−6 ± 25	0.78 (0.67, 0.85)	84 ± 33	82 ± 33	−2 ± 21	0.79 (0.69, 0.86)
	95 (52)	94 (62)			83 (42)	78 (39)		
Dietary fibre (g)	20.4 ± 9.5	18.8 ± 8.1	−1.7 ± 6.0 *	0.74 (0.70, 0.87)	17.4 ± 6.2	17.0 ± 7.1	−0.4 ± 4.5	0.81 (0.73, 0.88)
	18.2 (13.0)	17.1 (10.4)			17.5 (8.2)	15.3 (11.5)		
Total fat (g)	81.0 ± 44.5	79.6 ± 40.1	−1.4 ± 28.8	0.75 (0.62, 0.83)	62.6 ± 24.9	62.5 ± 27.6	−0.1 ± 20.5	0.69 (0.55, 0.79)
	67.9 (39.4)	70.9 (37.7)			56.5 (30.4)	57 (31.3)		
Saturated fat (g)	25.8 ± 13.9	26.1 ± 12.6	0.3 ± 8.9	0.68 (0.53, 0.79)	20.4 ± 9.5	20.2 ± 9.5	−0.2 ± 7.3	0.66 (0.51, 0.76)
	24.5 (14.8)	23.3 (15.6)			18.9 (10.5)	18.1 (10.8)		
Monounsaturated fat (g)	32.8 ± 18.5	32.3 ± 17.3	−0.4 ± 12.5	0.72 (0.59, 0.82)	25.2 ± 10.3	25.4 ± 11.8	0.2 ± 8.5	0.72 (0.59, 0.81)
	26.8 (16.3)	28.7 (14.8)			23.2 (11.2)	22.8 (12.9)		
Polyunsaturated fat (g)	15.7 ± 10.1	14.9 ± 8.9	−0.8 ± 6.7	0.68 (0.54, 0.79)	11.9 ± 5.0	11.8 ± 5.6	−0.1 ± 4.8	0.56 (0.39, 0.69)
	12.3 (9.7)	13.3 (7.3)			11.3 (5.5)	10.7 (5.6)		
Protein (g)	85.2 ± 46.7	82.4 ± 37.4	−2.8 ± 23.9	0.78 (0.68, 0.86)	65.1 ± 24.0	65.0 ± 26.8	−0.1 ± 19.8	0.69 (0.56, 0.79)
	74.1 (46.1)	71.4 (46.5)			61.9 (33.2)	58.0 (31.3)		
Cholesterol (g)	276 ± 197	260 ± 120	−15.4 ± 148.6	0.52 (0.33, 0.67)	208 ± 94	226 ± 123	18 ± 92.7	0.69 (0.57, 0.79)
	220 (172)	246 (171)			179 (117)	202 (101)		
Alcohol (g)	10.8 ± 14.3	9.5 ± 12.4	−1.3 ± 8.0	0.82 (0.72, 0.88)	5.1 ± 6.7	5.0 ± 5.5	−0.1 ± 4.0	0.79 (0.70, 0.86)
	5.3 (15.5)	3.9 (13.2)			2.2 (5.6)	2.3 (5.2)		
Caffeine (mg)	362 ± 248	321 ± 232	−41 ± 134 *	0.84 (0.75, 0.89)	242 ± 204	255 ± 227	12 ± 135	0.81 (0.73, 0.87)
	410 (271)	399 (268)			176 (373)	186 (377)		
Sodium (mg)	3055 ± 1558	2807 ± 1214	−248 ± 981 *	0.61 (0.44, 0.73)	2254 ± 806	2245 ± 944	−9 ± 669	0.66 (0.52, 0.77)
	2584 (1602)	2571 (1517)			2081 (1110)	2126 (863)		
Iron (mg)	14.8 ± 6.5	13.6 ± 5.2	−1.2 ± 4.1 *	0.62 (0.46, 0.74)	11.3 ± 3.6	11.1 ± 3.8	−0.2 ± 3.1	0.64 (0.49, 0.75)
	13.6 (7.9)	12.7 (5.6)			10.8 (5.0)	10.7 (5.5)		
Calcium (mg)	1037 ± 535	1011 ± 441	−27 ± 334	0.67 (0.52, 0.78)	910 ± 459	881 ± 504	−29 ± 328	0.79 (0.70, 0.86)
	957 (558)	961 (547)			796 (486)	727 (487)		
Vitamin D (mcg)	6.5 ± 3.5	6.4 ± 3.4	−0.1 ± 2.6	0.68 (0.53, 0.79)	5.3 ± 3.6	5.2 ± 3.8	−0.1 ± 2.4	0.80 (0.70, 0.86)
	6.2 (5.5)	5.7 (4.5)			4.2 (4.1)	4.4 (3.7)		
Total folate (mcg)	383 ± 222	341 ± 163	−42 ± 144 *	0.73 (0.61, 0.82)	321 ± 132	298 ± 115	−23 ± 99 *	0.62 (0.47, 0.74)
	314 (222)	299 (179)			297 (170)	277 (153)		
Vitamin B_12_ (mcg)	5.0 ± 2.9	4.7 ± 2.2	−0.3 ± 1.7	0.57 (0.39, 070)	3.8 ± 2.0	3.7 ± 2.0	−0.1 ± 1.4	0.73 (0.61, 0.82)
	4.7 (2.7)	4.1 (3.1)			3.5 (2.8)	3.4 (1.9)		

CDHQ-II: Canadian Diet History Questionnaire II, CI: Confidence Interval; ICC: Intra-class Correlation Coefficient, IQR: Interquartile Range, N/A: Not Applicable, SD: Standard Deviation. ^a^ Food and beverage sources only, excluding supplement sources; ^b^ ICC calculated for adjusted nutrients, measures similarity between Collection 1 and Collection 3 and ICC ≥ 0.60 indicates good similarity. Nutrient estimates were adjusted for total energy intake using the residual approach [27]; * *p* < 0.05 indicates difference in the means between Collection 1 and Collection 3 is significantly different from zero (statistical significance has been evaluated using either the Paired *t*-test or the Wilcoxon Signed-Rank test depending on normality assumption).

**Table 3:** Summary of energy and nutrients ^a^ for the two web CDHQ-II completions obtained at Collections 1 and 3 in the Web-Paper-Web group, for men and women (*n* = 127).

Nutrient	Men (*n* = 60)				Women (*n* = 67)			
	Mean ± SD, Median (IQR)		Mean Difference ± SD	ICC ^b^ (95% CI)	Mean ± SD, Median (IQR)		Mean Difference ± SD	ICC ^b^ (95% CI)
	Web Collection 1	Web Collection 3			Web Collection 1	Web Collection 3		
Total energy (kcal)	2429 ± 1033	2417 ± 1298	−12 ± 707	N/A	1521 ± 641	1411 ± 580	−110 ± 428 *	N/A
	2341 (1098)	2254 (1135)			1422 (657)	1323 (756)		
% energy carbohydrates	47.8 ± 6.5	47.3 ± 7.6	−0.5 ± 4.6	N/A	47.9 ± 10.0	47.1 ± 7.4	−0.8 ± 6.0	N/A
	47.6 (9.1)	46.6 (8.8)			48.2 (10.5)	47.5 (11.0)		
% energy total fat	34.5 ± 5.9	34.6 ± 5.9	0.1 ± 4.1	N/A	35.7 ± 6.8	35.8 ± 6.0	0.1 ± 5.0	N/A
	35.4 (7.7)	35.0 (6.6)			35.0 (8.3)	35.6 (7.2)		
% energy protein	17.0 ± 2.3	16.9 ± 3.1	−0.1 ± 2.3	N/A	16.4 ± 3.0	16.5 ± 2.9	0.1 ± 2.1	N/A
	16.6 (2.8)	16.4 (4.1)			16.4 (4.1)	16.4 (3.4)		
Carbohydrates (g)	289 ± 134	286 ± 175	−3 ± 91	0.83 (0.73, 0.89)	182 ± 79	168 ± 75	−14 ± 49 *	0.58 (0.40, 0.72)
	278 (138)	268 (135)			163 (87)	163 (120)		
Total sugars (g)	127 ± 81	123 ± 90	−4 ± 39	0.83 (0.74, 0.89)	79 ± 36	74 ± 37	−5 ± 21	0.58 (0.39, 0.72)
	115 (49)	109 (56)			73 (53)	67 (58)		
Dietary fibre (g)	25.9 ± 10.8	25.8 ± 16.2	−0.1 ± 9.9	0.81 (0.70, 0.88)	18.1 ± 8.7	16.1 ± 8.2	−2.0 ± 4.6 ^†^	0.79 (0.68, 0.86)
	25.7 (12.4)	23.4 (12.6)			16.9 (9.7)	13.9 (10.5)		
Total fat (g)	93.8 ± 42.7	94.1 ± 53.6	0.3 ± 29.0	0.79 (0.68, 0.87)	60.7 ± 30.1	56.5 ± 27.5	−4.3 ± 21.0	0.66 (0.50, 0.77)
	88.0 (51.2)	81.5 (53.7)			55.4 (32.9)	51.0 (23.8)		
Saturated fat (g)	31.5 ± 16.9	31.1 ± 17.1	−0.4 ± 8.3	0.80 (0.69, 0.88)	19.1 ± 9.6	18.4 ± 9.4	−0.8 ± 6.4	0.68 (0.53, 0.79)
	27.4 (16.4)	27.2 (18.7)			17.4 (8.7)	17.0 (9.1)		
Monounsaturated fat (g)	37.4 ± 16.9	37.8 ± 22.9	0.4 ± 13.4	0.74 (0.60, 0.83)	24.7 ± 12.4	22.8 ± 10.8	−1.9 ± 9.2	0.55 (0.36, 0.70)
	36.7 (20.9)	31.9 (24.2)			21.9 (12.9)	21.1 (12.1)		
Polyunsaturated fat (g)	17.2 ± 7.7	17.7 ± 11.8	0.5 ± 7.8	0.54 (0.34, 0.70)	11.7 ± 7.0	10.5 ± 6.0	−1.1 ± 4.6 *	0.74 (0.61, 0.83)
	16.3 (8.7)	15.7 (11.2)			10.1 (8.5)	9.8 (5.4)		
Protein (g)	101.8 ± 41.9	101.3 ± 52.5	−0.5 ± 29.7	0.65 (0.48, 0.78)	63.2 ± 33.1	57.9 ± 23.3	−5.3 ± 25.5	0.61 (0.44, 0.74)
	97.5 (43.8)	96.9 (52.0)			58.1 (35.7)	55.1 (33.7)		
Cholesterol (g)	312 ± 142	307 ± 172	−5 ± 114	0.71 (0.56, 0.81)	193 ± 114	186 ± 83	−8 ± 83	0.71 (0.57, 0.81)
	292 (171)	278 (167)			174 (84)	180 (95)		
Alcohol (g)	11.0 ± 14.4	10.9 ± 15.3	−0.1 ± 12.7	0.65 (0.48, 0.78)	5.0 ± 7.4	4.9 ± 6.9	−0.1 ± 2.6	0.93 (0.89, 0.95)
	6.2 (15.3)	6.7 (13.6)			1.7 (5.6)	1.9 (5.6)		
Caffeine (mg)	289 ± 244	281 ± 266	−8 ± 125	0.87 (0.80, 0.92)	267.5 ± 215	268 ± 222	0.5 ± 102.1	0.89 (0.84, 0.93)
	236 (388)	188 (339)			308 (342)	242 (342)		
Sodium (mg)	3453 ± 1525	3484 ± 2389	31 ± 1316	0.59 (0.39, 0.73)	2229 ± 1051	2067 ± 904	−161 ± 762	0.48 (0.27, 0.64)
	3287 (1878)	3001 (1943)			2016 (1289)	1924 (1274)		
Iron (mg)	17.6 ± 6.6	17.9 ± 10.6	0.3 ± 6.4	0.67 (0.50, 0.79)	11.4 ± 4.6	10.2 ± 4.0	−1.2 ± 3.3 ^†^	0.61 (0.44, 0.74)
	17.2 (7.6)	17.3 (7.8)			10.8 (5.1)	9.8 (5.4)		
Calcium (mg)	1338 ± 905	1185 ± 576	−153 ± 550 *	0.44 (0.22, 0.62)	764 ± 373	706 ± 367	−58 ± 235 *	0.62 (0.45, 0.75)
	1158 (661)	1157 (606)			643 (506)	619 (431)		
Vitamin D (mcg)	8.4 ± 7.4	7.7 ± 6.3	−0.7 ± 3.0	0.74 (0.60, 0.83)	4.1 ± 2.4	4.0 ± 2.6	−0.1 ± 1.7	0.78 (0.67, 0.86)
	7.1 (4.4)	6.6 (4.9)			3.2 (2.6)	3.2 (2.5)		
Total folate (mcg)	446 ± 219	447 ± 345	1 ± 192	0.75 (0.61, 0.84)	312 ± 145	278 ± 134	−34 ± 83 ^†^	0.75 (0.62, 0.84)
	433 (202)	415 (253)			305 (170)	279 (179)		
Vitamin B_12_ (mcg)	6.4 ± 3.7	6.9 ± 8.8	0.5 ± 6.8	0.26 (0.01, 0.48)	3.5 ± 1.9	3.4 ± 1.8	−0.1 ± 1.6	0.66 (0.50, 0.77)
	5.7 (3.8)	5.7 (3.4)			3.1 (2.4)	3.0 (2.2)		

CDHQ-II: Canadian Diet History Questionnaire II, CI: Confidence Interval; ICC: Intra-class Correlation Coefficient, IQR: Interquartile Range, N/A: Not Applicable, SD: Standard Deviation. ^a^ Food and beverage sources only, excluding supplement sources; ^b^ ICC calculated for adjusted nutrients, measures similarity between Collection 1 and Collection 3 and ICC ≥ 0.60 indicates good similarity. Nutrient estimates were adjusted for total energy intake using the residual approach [27]; * *p* < 0.05, ^†^ *p* < 0.01 indicates difference in the means between Collection 1 and Collection 3 is significantly different from zero (statistical significance has been evaluated using either the Paired *t*-test or the Wilcoxon Signed-Rank test depending on normality assumption).

**Table 4:** Overall comparison of energy and nutrients ^a^ for paper and web CDHQ-II (*n* = 347) ^b^.

Nutrient	Men (*n* = 176)				Women (*n* = 171)			
	Mean ± SD, Median (IQR)		Mean Difference ± SD	ICC ^c^ (95% CI)	Mean ± SD, Median (IQR)		Mean Difference ± SD	ICC ^c^ (95% CI)
	Paper Overall	Web Overall			Paper Overall	Web Overall		
Total energy (kcal)	2145 ± 926	2149 ± 963	4 ± 469	N/A	1465 ± 513	1523 ± 538	58 ± 340 *	N/A
	1938 (1083)	2020 (1176)			1384 (576)	1498 (725)		
% energy carbohydrates	47.0 ± 7.5	46.6 ± 7.2	−0.4 ± 4.6	N/A	46.9 ± 6.7	47.4 ± 6.9	0.5 ± 5.2	N/A
	46.6 (10.1)	46.8 (8.7)			47.2 (9.1)	47.7 (9.3)		
% energy total fat	35.1 ± 6.0	35.0 ± 6.0	−0.1 ± 3.9	N/A	36.1 ± 5.6	36.0 ± 5.8	−0.1 ± 4.3	N/A
	34.8 (7.3)	34.8 (6.5)			36.2 (7.0)	35.9 (6.7)		
% energy protein	16.6 ± 2.8	16.7 ± 2.7	0.1 ± 2.0	N/A	16.9 ± 3.0	16.7 ± 2.9	−0.2 ± 1.9	N/A
	16.1 (3.4)	16.6 (3.1)			16.8 (3.7)	16.6 (3.6)		
Carbohydrates (g)	250 ± 120	249 ± 121	−1 ± 52	0.80 (0.74, 0.85)	172 ± 66	180 ± 67	8 ± 49 *	0.60 (0.49, 0.69)
	232 (135)	229 (137)			164 (84)	171 (94)		
Total sugars (g)	111 ± 69	108 ± 63	−3 ± 25	0.84 (0.79, 0.88)	79 ± 35	79 ± 34	<1 ± 21	0.71 (0.63, 0.78)
	97 (51)	101 (55)			75 (49)	77 (41)		
Dietary fibre (g)	21.8 ± 11.1	21.7 ± 11.4	−0.1 ± 6.1	0.80 (0.74, 0.85)	16.5 ± 6.7	17.4 ± 7.5	1.0 ± 4.5 *	0.81 (0.75, 0.85)
	19.8 (12.7)	20.5 (13.4)			15.6 (9.9)	16.3 (9.5)		
Total fat (g)	84.4 ± 40.6	83.8 ± 41.0	−0.6 ± 24.0	0.76 (0.69, 0.82)	59.3 ± 24.5	61.5 ± 25.9	2.2 ± 16.8	0.66 (0.56, 0.73)
	76.3 (49.2)	76.8 (50.9)			55.1 (24.8)	58.7 (30.0)		
Saturated fat (g)	27.3 ± 13.5	27.8 ± 14.1	0.5 ± 7.9	0.78 (0.72, 0.83)	18.9 ± 8.3	19.5 ± 8.7	0.6 ± 5.9	0.65 (0.56, 0.73)
	24.4 (16.4)	25.1 (16.4)			17.4 (8.5)	18.2 (8.4)		
Monounsaturated fat (g)	34.2 ± 16.9	33.8 ± 16.8	−0.5 ± 10.2	0.75 (0.68, 0.81)	24.0 ± 10.2	25.1 ± 10.9	1.1 ± 7.1 *	0.67 (0.57, 0.74)
	30.4 (23.4)	29.8 (19.7)			22.7 (10.5)	24.4 (12.8)		
Polyunsaturated fat (g)	15.9 ± 8.8	15.5 ± 8.9	−0.4 ± 5.3	0.77 (0.70, 0.82)	11.4 ± 5.4	11.8 ± 5.6	0.4 ± 3.8	0.71 (0.63, 0.78)
	13.2 (10.3)	14.0 (9.8)			10.6 (6.6)	11.2 (7.0)		
Protein (g)	89.3 ± 40.9	90.1 ± 43.1	0.8 ± 22.7	0.72 (0.64, 0.78)	61.6 ± 23.3	63.7 ± 26.2	2.2 ± 14.1 *	0.78 (0.71, 0.83)
	80.2 (50.2)	84.8 (51.1)			58.2 (27.5)	59.8 (31.6)		
Cholesterol (g)	283 ± 144	280 ± 139	−3 ± 87	0.78 (0.71, 0.83)	201 ± 99	199 ± 94	−2 ± 57	0.77 (0.71, 0.83)
	254 (195)	263 (167)			179 (104)	187 (114)		
Alcohol (g)	10.8 ± 14.7	12.0 ± 19.2	1.2 ± 13.3	0.70 (0.61, 0.76)	4.7 ± 6.1	4.5 ± 6.2	−0.2 ± 4.1	0.78 (0.72, 0.83)
	5.6 (13.1)	5.5 (15.4)			1.9 (5.4)	2.0 (5.4)		
Caffeine (mg)	313 ± 233	325 ± 249	12 ± 133	0.84 (0.79, 0.88)	254 ± 218	239 ± 203	−14 ± 139	0.79 (0.73, 0.84)
	313 (310)	327 (287)			210 (369)	196 (366)		
Sodium (mg)	3084 ± 1397	3081 ± 1571	−3 ± 885	0.61 (0.51, 0.69)	2163 ± 829	2228 ± 880	65 ± 546	0.65 (0.56, 0.73)
	2667 (1660)	2755 (1587)			2062 (971)	2159 (1219)		
Iron (mg)	15.4 ± 6.7	15.2 ± 7.1	−0.2 ± 3.8	0.71 (0.63, 0.77)	10.8 ± 3.8	11.2 ± 4.0	0.4 ± 2.6	0.72 (0.65, 0.79)
	14.3 (8.5)	14.8 (8.7)			10.2 (4.9)	10.9 (5.2)		
Calcium (mg)	1083 ± 588	1095 ± 606	12 ± 278	0.82 (0.77, 0.87)	837 ± 452	838 ± 440	1 ± 270	0.81 (0.75, 0.85)
	953 (544)	1008 (559)			707 (532)	744 (484)		
Vitamin D (mcg)	6.8 ± 4.3	6.9 ± 5.0	0.1 ± 2.4	0.77 (0.70, 0.82)	4.8 ± 3.1	4.8 ± 3.3	0.1 ± 1.5	0.84 (0.79, 0.88)
	5.8 (4.4)	6.1 (4.1)			3.4 (3.6)	3.9 (2.8)		
Total folate (mcg)	388 ± 207	382 ± 220	−7 ± 135	0.63 (0.54, 0.71)	306 ± 146	315 ± 148	9 ± 98	0.79 (0.72, 0.84)
	349 (219)	354 (228)			277 (152)	303 (172)		
Vitamin B_12_ (mcg)	5.4 ± 3.2	5.6 ± 4.2	0.1 ± 3.2	0.30 (0.16, 0.43)	3.8 ± 2.4	3.7 ± 1.2	−0.1 ± 1.6	0.59 (0.49, 0.68)
	4.8 (2.9)	5.0 (3.4)			3.4 (2.5)	3.3 (2.2)		

CDHQ-II: Canadian Diet History Questionnaire II, CI: Confidence Interval; ICC: Intra-class Correlation Coefficient, IQR: Interquartile Range, N/A: Not Applicable, SD: Standard Deviation. ^a^ Food and beverage sources only, excluding supplement sources; ^b^ 66 Participants who completed the first two questionnaires at Collections 1 and 2 but not at Collection 3, were included in this inter-version reliability analysis, hence the total number of participants in this table is higher than the total in Table 2 and Table 3; ^c^ ICC calculated for adjusted nutrients, measures similarity between paper and web and ICC ≥ 0.60 indicates good similarity. Nutrient estimates were adjusted for total energy intake using the residual approach [27]; * *p* < 0.05 indicates difference in the means between paper and web is significantly different from zero (statistical significance has been evaluated using either the Paired *t*-test or the Wilcoxon Signed-Rank test depending on normality assumption).

**Table 5:** Time spent and future willingness to complete a CDHQ-II online by browser type, internet connection and operating system.

	Participants (%) ^a^			Time Spent (Minutes) ^b^			Future Willingness to Complete CDHQ-II Online (%) ^c^		
	Paper-Web-Paper Collection 2	Web-Paper-Web Collection 1	Web-Paper-Web Collection 3	Paper-Web-Paper Collection 2	Web-Paper-Web Collection 1	Web-Paper-Web Collection 3	Paper-Web-Paper Collection 2	Web-Paper-Web Collection 1	Web-Paper-Web Collection 3
**Browser**	(*n* = 165)	(*n* = 173)	(*n* = 126)	(*n* = 156)	(*n* = 168)	(*n* = 116)	(*n* = 157)	(*n* = 171)	(*n* = 121)
Internet Explorer	81 (49.1)	77 (44.5)	55 (43.7)	79.3 ± 33.2	73.8 ± 30.6	76.1 ± 32.1	69 (92.0)	70 (90.9)	49 (89.1)
Safari	31 (18.8)	31 (17.9)	21 (16.7)	87.7 ± 51.1	78.4 ± 34.3	71.3 ± 24.9	30 (96.8)	30 (96.8)	20 (100.0)
Google Chrome	36 (21.8)	50 (28.9)	38 (30.2)	78.2 ± 30.8	81.2 ± 37.9	76.2 ± 28.1	32 (94.1)	47 (97.9)	33 (91.7)
Mozilla Firefox	17 (10.3)	15 (8.7)	12 (9.5)	79.7 ± 27.0	86.1 ± 41.5	72.3 ± 26.1	17 (100.0)	15 (100.0)	10 (90.9)
Opera	0 (0.0)	0 (0.0)	0 (0.0)	N/A	N/A	N/A	N/A	N/A	N/A
Other	0 (0.0)	0 (0.0)	0 (0.0)	N/A	N/A	N/A	N/A	N/A	N/A
**Internet Connection**	(*n* = 156)	(*n* = 165)	(*n* = 121)	(*n* = 147)	(*n* = 160)	(*n* = 111)	(*n* = 148)	(*n* = 163)	(*n* = 116)
Low speed ^d^	20 (12.8)	17 (10.3)	13 (10.7)	78.5 ± 26.1	97.9 ± 50.3	78.8 ± 45.1	17 (94.4)	16 (94.1)	11 (84.6)
Medium speed ^e^	34 (21.8)	42 (25.5)	30 (24.8)	90.8 ± 39.5	77.4 ± 26.8	73.8 ± 19.7	31 (96.9)	37 (92.5)	26 (92.9)
High speed ^f^	102 (65.4)	106 (64.2)	78 (64.5)	78.4 ± 37.6	74.2 ± 31.7	74.2 ± 28.5	91 (92.9)	101 (95.3)	70 (93.3)
**Operating System**	(*n* = 164)	(*n* = 170)	(*n* = 126)	(*n* = 155)	(*n* = 165)	(*n* = 116)	(*n* = 156)	(*n* = 168)	(*n* = 121)
Windows	125 (76.2)	135 (79.4)	98 (77.8)	82.7 ± 49.6	75.7 ± 32.3	72.7 ± 25.5	111 (94.9)	125 (94.0)	85 (90.4)
MAC OS	35 (21.3)	35 (20.5)	27 (21.4)	79.5 ± 31.3	78.1 ± 35.1	74.7 ± 29.7	32 (91.4)	35 (100.0)	26 (100.0)
Other	4 (2.4)	0 (0.0)	1 (0.8)	106.3 ± 43.1	N/A	60.0 *	4 (100.0)	N/A	1 (100.0)

CDHQ-II: Canadian Diet History Questionnaire II, N/A: Not Applicable. ^a^ Column percentages; ^b^ Values are expressed as mean ± standard deviation; ^c^ Row percentages; ^d^ Low speed internet includes: dial-up, mobile 3G, and Integrated Services Digital Network; ^e^ Medium speed internet includes: public Wi-Fi, mobile 4G LTE, and satellite, ^f^ High speed internet includes: Digital Subscriber Line, and cable Modem. * There is no standard deviation provided as there was only one respondent for this collection in this category.

## Supplementary Materials

The following are available online at http://www.mdpi.com/2072-6643/9/2/133/s1; Table S1: Summary of energy and nutrients for the two paper CDHQ-II and two web CDHQ-II completions at Collections 1 and 3, and overall paper and web CDHQ-II, for men and women; Table S2: Agreement in dietary supplements for the two paper CDHQ-II and two web CDHQ-II completions at Collections 1 and 3, and overall paper and web CDHQ-II; Table S3: Time and group effects for energy and nutrients; Table S4: Future willingness to complete CDHQ-II online by socio-demographic characteristics.
