# Supplementary material for: The Comparative Reliability and Feasibility of the Past-Year Canadian Diet History Questionnaire II: Comparison of the Paper and Web Versions
Source: Nutrients. 2017 Feb 13;9(2):133. doi: 10.3390/nu9020133 (PMC5331564; doi:10.3390/nu9020133)
Supplement: Supplementary file 1 [file nutrients-09-00133-s001.docx]

Supplementary Materials: The Comparative Reliability and Feasibility of the Past-Year Canadian Diet History Questionnaire II: Comparison of the Paper and Web Versions

Geraldine Lo Siou, Ilona Csizmadi, Beatrice A. Boucher, Alianu K. Akawung, Heather K. Whelan, Michelle Sharma, Ala Al Rajabi, Jennifer E. Vena, Sharon I. Kirkpatrick, Anita Koushik, Isabelle Massarelli, Isabelle Rondeau and Paula J. Robson

**Table S1.** Summary of energy and nutrients ^a^ for the two paper CDHQ-II and two web CDHQ-II completions at Collections 1 and 3, and overall paper and web CDHQ-II, for men and women.

| **Nutrient** | **Unadjusted ICC ^b^ (95% CI)** | | | | | |
| --- | --- | --- | --- | --- | --- | --- |
|  | **Paper Collection 1 vs. Paper Collection 3** | | **Web Collection 1 vs. Web Collection 3** | | **Paper vs. Web Overall ^c^** | |
|  | **Men (*n* = 72)** | **Women (*n* = 82)** | **Men (*n* = 60)** | **Women (*n* = 67)** | **Men (*n* = 176)** | **Women (*n* = 171)** |
| Total energy (kcal) | 0.80 (0.69, 0.87) | 0.69 (0.55, 0.79) | 0.82 (0.71, 0.89) | 0.76 (0.63, 0.84) | 0.89 (0.84, 0.91) | 0.79 (0.73, 0.84) |
| % energy carbohydrates | 0.80 (0.70, 0.87) | 0.78 (0.68, 0.85) | 0.79 (0.67, 0.87) | 0.70 (0.56, 0.81) | 0.80 (0.74, 0.85) | 0.71 (0.62, 0.78) |
| % energy total fat | 0.74 (0.62, 0.83) | 0.76 (0.65, 0.84) | 0.77 (0.64, 0.85) | 0.70 (0.55, 0.80) | 0.79 (0.72, 0.84) | 0.72 (0.64, 0.78) |
| % energy protein | 0.75 (0.62, 0.83) | 0.75 (0.64, 0.83) | 0.63 (0.45, 0.76) | 0.76 (0.63, 0.84) | 0.72 (0.65, 0.79) | 0.79 (0.72, 0.84) |
| Carbohydrates (g) | 0.79 (0.68, 0.86) | 0.70 (0.57, 0.80) | 0.83 (0.73, 0.90) | 0.80 (0.70, 0.87) | 0.91 (0.88, 0.93) | 0.73 (0.65, 0.80) |
| Total sugars (g) | 0.81 (0.71, 0.88) | 0.80 (0.71, 0.87) | 0.90 (0.83, 0.94) | 0.84 (0.75, 0.90) | 0.93 (0.90, 0.95) | 0.81 (0.75, 0.85) |
| Dietary fiber (g) | 0.87 (0.79, 0.92) | 0.88 (0.80, 0.92) | 0.74 (0.61, 0.84) | 0.85 (0.77, 0.91) | 0.85 (0.81, 0.89) | 0.80 (0.74, 0.85) |
| Total fat (g) | 0.77 (0.66, 0.85) | 0.70 (0.57, 0.79) | 0.82 (0.72, 0.89) | 0.73 (0.60, 0.83) | 0.83 (0.77, 0.87) | 0.78 (0.71, 0.83) |
| Saturated fat (g) | 0.77 (0.66, 0.85) | 0.70 (0.57, 0.80) | 0.88 (0.81, 0.93) | 0.77 (0.66, 0.86) | 0.84 (0.79, 0.88) | 0.76 (0.69, 0.82) |
| Monounsaturated fat (g) | 0.76 (0.64, 0.84) | 0.71 (0.58, 0.80) | 0.78 (0.66, 0.86) | 0.68 (0.53, 0.79) | 0.82 (0.76, 0.86) | 0.78 (0.71, 0.83) |
| Polyunsaturated fat (g) | 0.75 (0.63, 0.84) | 0.60 (0.44, 0.72) | 0.69 (0.53, 0.80) | 0.76 (0.63, 0.84) | 0.82 (0.76, 0.86) | 0.76 (0.69, 0.82) |
| Protein (g) | 0.84 (0.76, 0.90) | 0.70 (0.57, 0.79) | 0.81 (0.69, 0.88) | 0.60 (0.43, 0.74) | 0.85 (0.81, 0.89) | 0.84 (0.79, 0.88) |
| Cholesterol (g) | 0.59 (0.41, 0.72) | 0.64 (0.49, 0.75) | 0.74 (0.60, 0.84) | 0.66 (0.50, 0.78) | 0.81 (0.75, 0.86) | 0.83 (0.77, 0.87) |
| Alcohol (g) | 0.82 (0.73, 0.88) | 0.79 (0.69, 0.86) | 0.64 (0.46, 0.77) | 0.94 (0.90, 0.96) | 0.70 (0.61, 0.77) | 0.77 (0.71, 0.83) |
| Caffeine (mg) | 0.84 (0.76, 0.90) | 0.81 (0.71, 0.87) | 0.88 (0.81, 0.93) | 0.89 (0.83, 0.93) | 0.85 (0.80, 0.89) | 0.78 (0.72, 0.83) |
| Sodium (mg) | 0.75 (0.63, 0.84) | 0.71 (0.58, 0.80) | 0.78 (0.66, 0.87) | 0.70 (0.55, 0.80) | 0.82 (0.77, 0.87) | 0.80 (0.73, 0.85) |
| Iron (mg) | 0.76 (0.64, 0.84) | 0.66 (0.52, 0.77) | 0.74 (0.59, 0.83) | 0.70 (0.56, 0.81) | 0.85 (0.80, 0.88) | 0.78 (0.72, 0.83) |
| Calcium (mg) | 0.77 (0.65, 0.85) | 0.77 (0.66, 0.84) | 0.74 (0.60, 0.83) | 0.80 (0.69, 0.87) | 0.89 (0.86, 0.92) | 0.82 (0.76, 0.86) |
| Vitamin D (mcg) | 0.71 (0.57, 0.81) | 0.80 (0.70, 0.86) | 0.90 (0.84, 0.94) | 0.76 (0.63, 0.84) | 0.87 (0.83, 0.90) | 0.90 (0.86, 0.92) |
| Total folate (mcg) | 0.73 (0.59, 0.82) | 0.68 (0.54, 0.78) | 0.78 (0.65, 0.86) | 0.82 (0.73, 0.89) | 0.80 (0.74, 0.85) | 0.78 (0.71, 0.83) |
| Vitamin B_12_ (mcg) | 0.77 (0.65, 0.85) | 0.75 (0.64, 0.83) | 0.49 (0.27, 0.66) | 0.64 (0.48, 0.76) | 0.63 (0.54, 0.71) | 0.73 (0.65, 0.79) |

CDHQ-II: Canadian Diet History Questionnaire II, CI: Confidence Interval; ICC: Intra-class Correlation Coefficients. ^a^ Food and beverage sources only, excluding supplement sources; ^b^ ICC calculated for unadjusted nutrients, measures similarity between Collection 1 and Collection 3 and overall; ICC ≥0.60 indicates good similarity;
^c^ 66 Participants who completed the first two questionnaires at Collections 1 and 2 but not at Collection 3, were included in this inter-version reliability analysis, hence the total number of participants in this third column (Paper vs. Web Overall) is higher than the totals in the first two columns (Paper Collection 1 vs. Paper Collection 3; Web Collection 1 vs. Web Collection 3).

**Table S2.** Agreement in dietary supplements for the two paper CDHQ-II and two web CDHQ-II completions at Collections 1 and 3, and overall paper and web CDHQ-II.

| **Dietary Supplements** | **Kappa Measure of Agreement** | | |
| --- | --- | --- | --- |
|  | **Paper Collection 1 vs. Paper Collection 3 (*n* = 154)** | **Web Collection 1 vs. Web Collection 3 (*n* = 127)** | **Paper vs. Web Overall (*n* = 347)** |
| Vitamin A | 0.8 | 0.9 | 0.8 |
| Beta carotene | 0.4 | 0.8 | 0.6 |
| Vitamin E | 0.7 | 0.8 | 0.8 |
| Vitamin C | 0.7 | 0.8 | 0.7 |
| Thiamin | 0.7 | 0.8 | 0.8 |
| Riboflavin | 0.7 | 0.8 | 0.8 |
| Niacin | 0.7 | 0.8 | 0.8 |
| Vitamin B_6_ | 0.7 | 0.8 | 0.8 |
| Folic acid | 0.7 | 0.8 | 0.8 |
| Vitamin B_12_ | 0.7 | 0.8 | 0.8 |
| Calcium | 0.8 | 0.8 | 0.8 |
| Magnesium | 0.7 | 0.8 | 0.8 |
| Iron | 0.8 | 0.8 | 0.8 |
| Zinc | 0.7 | 0.7 | 0.7 |
| Copper | 0.8 | 0.9 | 0.8 |
| Vitamin D | 0.7 | 0.8 | 0.8 |
| Selenium | 0.8 | 0.8 | 0.8 |
| Pantothenic acid | 0.7 | 0.8 | 0.8 |
| Lutein | 0.7 | 0.9 | 0.8 |
| Potassium | 0.8 | 0.9 | 0.8 |
| Manganese | 0.8 | 0.8 | 0.8 |
| Lycopene | 0.7 | 0.9 | 0.8 |
| Vitamin K | 0.7 | 0.9 | 0.8 |
| Phosphate | 0.8 | 0.9 | 0.8 |
| Folic acid | 0.7 | 0.8 | 0.8 |
| Vitamin E | 0.7 | 0.9 | 0.8 |
| Calcium antacid | 0.7 | 0.7 | 0.4 |

CDHQ-II: Canadian Diet History Questionnaire II.

**Table S3.** Time and group effects for energy and nutrients ^a^ (*n* = 268).

| **Nutrient** | **Main Effect of Collection** | **Main Effect of Group** | **Interaction Effect between Collection and Group** |
| --- | --- | --- | --- |
| Total energy (kcal) | *F*(2, 524) = 0.494, *p* = 0.611 | *F*(1, 262) = 1.291, *p* = 0.257 | *F*(2, 524) = 0.820, *p* = 0.441 |
| % energy carbohydrates | *F*(2, 524) = 0.516, *p* = 0.597 | *F*(1, 262) = 0.309, *p* = 0.579 | *F*(2, 524) = 0.340, *p* = 0.712 |
| % energy total fat | *F*(2, 524) = 0.679, *p =* 0.508 | *F*(1, 262) = 0.397, *p =* 0.529 | *F*(2, 524) = 0.669, *p* = 0.513 |
| % energy protein | *F*(2, 524) = 0.245, *p =* 0.783 | *F*(1, 262) = 0.469, *p* = 0.494 | *F*(2, 524) = 0.867, *p* = 0.421 |
| Carbohydrates (g) | *F*(2, 524) = 0.545, *p =* 0.580 | *F*(1, 262) = 2.121, *p* = 0.147 | *F*(2, 524) = 1.169, *p* = 0.312 |
| Total sugars (g) | *F*(2, 524) = 0.162, *p* = 0.850 | *F*(1, 262) = 1.268, *p* = 0.261 | *F*(2, 524) = 0.196, *p* = 0.822 |
| Dietary fiber (g) | *F*(2, 524) = 0.002, *p =* 0.999 | *F*(1, 262) = 4.134, *p* = 0.043* | *F*(2, 524) = 1.313, *p* = 0.270 |
| Total fat (g) | *F*(2, 524) = 0.840, *p* = 0.432 | *F*(1, 262) = 0.376, *p* = 0.540 | *F*(2, 524) = 0.263, *p* = 0.769 |
| Saturated fat (g) | *F*(2, 524) = 0.572, *p* = 0.565 | *F*(1, 262) = 0.553, *p* = 0.458 | *F*(2, 524) = 0.951, *p* = 0.387 |
| Monounsaturated fat (g) | *F*(2, 524) = 0.803, *p* = 0.448 | *F*(1, 262) = 0.276, *p* = 0.600 | *F*(2, 524) = 0.225, *p* = 0.799 |
| Polyunsaturated fat (g) | *F*(2, 524) = 0.759, *p* = 0.469 | *F*(1, 262) = 0.066, *p* = 0.797 | *F*(2, 524) = 0.054, *p* = 0.948 |
| Protein (g) | *F*(2, 524) = 0.726, *p* = 0.484 | *F*(1, 262) = 1.186, *p* = 0.277 | *F*(2, 524) = 0.838, *p* = 0.433 |
| Cholesterol (g) | *F*(2, 524) = 0.561, *p* = 0.571 | *F*(1, 262) = 0.355, *p =* 0.552 | *F*(2, 524) = 0.350, *p* = 0.705 |
| Alcohol (g) | *F*(2, 524) = 0.966, *p* = 0.381 | *F*(1, 262) = 0.033, *p* = 0.857 | *F*(2, 524) = 0.223, *p* = 0.800 |
| Caffeine (mg) | *F*(2, 524) = 0.229, *p* = 0.795 | *F*(1, 262) = 0.928, *p* = 0.336 | *F*(2, 524) = 0.749, *p* = 0.473 |
| Sodium (mg) | *F*(2, 524) = 0.418, *p* = 0.659 | *F*(1, 262) = 1.127, *p* = 0.289 | *F*(2, 524) = 0.417, *p* = 0.659 |
| Iron (mg) | *F*(2, 524) = 0.262, *p* = 0.770 | *F*(1, 262) = 3.571, *p* = 0.060 | *F*(2, 524) = 0.305, *p* = 0.737 |
| Calcium (mg) | *F*(2, 524) = 0.283, *p* = 0.753 | *F*(1, 262) = 0.000, *p* = 0.997 | *F*(2, 524) = 2.529, *p* = 0.081 |
| Vitamin D (mcg) | *F*(2, 524) = 1.851, *p* = 0.158 | *F*(1, 262) = 0.137, *p* = 0.711 | *F*(2, 524) = 0.927, *p* = 0.396 |
| Total folate (mcg) | *F*(2, 524) = 0.235, *p* = 0.790 | *F*(1, 262) = 1.446, *p* = 0.230 | *F*(2, 524) = 0.734, *p* = 0.480 |
| Vitamin B_12_ (mcg) | *F*(2, 524) = 0.941, *p* = 0.391 | *F*(1, 262) = 2.723, *p* = 0.100 | *F*(2, 524) = 0.809, *p* = 0.446 |

^a^ Food and beverage sources only, excluding supplement sources. * *p* < 0.05 indicates difference in the means between at least 2 collection points, between groups (Paper-Web-Paper vs. Web-Paper-Web) and between groups at each collection point is significantly different from zero (Statistical significance has been evaluated using the *F*-test in mixed Analysis of Variance (ANOVA)). Mixed ANOVA were adjusted for sex, age and body mass index.

**Table S4.** Future willingness to complete CDHQ-II online by to socio-demographic characteristics.

| **Future Willingness to** | **Paper-Web-Paper** | | | **Web-Paper-Web** | | |
| --- | --- | --- | --- | --- | --- | --- |
| **Complete CDHQ-II Online** | **Collection 1 (*n* = 210) %** | **Collection 2 (*n* = 158) %** | **Collection 3 (*n* = 133) %** | **Collection 1 (*n* = 172) %** | **Collection 2 (*n* = 169) %** | **Collection 3 (*n* = 124) %** |
| Total | 89.1 | 94.3 | 89.5 | 94.8 | 88.8 | 91.9 |
| Sex |  |  |  |  |  |  |
| Men | 86.1 | 94.8 | 93.6 | 93.0 | 88.4 | 89.8 |
| Women | 92.2 | 93.8 | 85.9 | 96.5 | 89.2 | 93.9 |
| Age (Years) |  |  |  |  |  |  |
| <55 | 89.9 | 94.5 | 89.5 | 95.1 | 90.1 | 90.7 |
| ≥55 | 88.3 | 94.1 | 89.5 | 94.5 | 87.5 | 92.9 |
| Educational attainment |  |  |  |  |  |  |
| Elementary school | 75.0 | 99.9 | 99.9 | 99.8 | 99.7 | 99.9 |
| High school completed | 83.7 | 86.1 | 87.1 | 90.7 | 92.5 | 88.5 |
| Some post-secondary ^a^ | 91.1 | 97.0 | 86.8 | 95.4 | 85.9 | 92.4 |
| Post-secondary completed ^b^ | 91.0 | 96.2 | 93.6 | 97.5 | 90.0 | 92.9 |
| Geographic location ^c^ |  |  |  |  |  |  |
| Urban | 92.9 | 96.3 | 89.6 | 97.7 | 94.0 | 93.3 |
| Rural | 85.6 | 92.1 | 89.4 | 91.7 | 83.7 | 90.6 |

CDHQ-II: Canadian Diet History Questionnaire II. ^a^ Some post-secondary includes: Trade, technical or vocational school, apprenticeship training or technical CEGEP, diploma from community college, pre-university CEGEP or non-university certificate, and university certificate below Bachelor’s level; ^b^ Post-secondary completed includes: Bachelor’s, and graduate degree (MSc, MBA, MD, PhD etc.); ^c^ Geographic location was based on Canada postal codes where the second digit was 0 for
rural regions.
